# Clinical characteristics and prognosis of 16 relapsed/refractory B-cell malignancy patients with CAR T-cell-related hyperferritinaemia

**DOI:** 10.3389/fonc.2022.912689

**Published:** 2022-10-12

**Authors:** Lanlan Zhou, Nanzhou Yu, Tongjuan Li, Hongyan Ji, Lijun Jiang, Di Wang, Bin Xu, Xiaoxi Zhou

**Affiliations:** ^1^ Department of Hematology, The First Affiliated Hospital of Guangdong Pharmaceutical University, Guangzhou, China; ^2^ Department of Hematology, Tongji Hospital, TongJi Medical College, Huazhong University of Science and Technology, Wuhan, China

**Keywords:** B-cell malignancies, CAR T-cell therapy, hyperferritinaemia, prognosis, clinical characteristics

## Abstract

With the success of chimeric antigen receptor-modified (CAR) T-cell therapy for relapsed/refractory (r/r) B-cell malignancies, severe complications after CAR T-cell infusion have emerged as nonnegligible prognosis-related factors. However, the prognosis of patients with CAR T-cell-related hyperferritinaemia (HFA) is unclear. We report the efficacy and safety of CAR T-cell therapy in 16 r/r B-cell malignancy patients with CAR T-cell-related HFA. The rates of serum ferritin levels above 10,000 ng/ml during CAR T-cell therapy were 6.2% and 14.3% in B-cell non-Hodgkin’s lymphoma (B-NHL) and acute B lymphocyte leukemia (B-ALL), respectively. These patients were characterized by an extremely high tumor burden and a high rate of extranodal involvement. In lymphoma, the complete remission (CR) rate was 37.5% (3/8), which was lower than that in the control group with the lowest value of ferritin (CR was 87.5% (7/8), P=0.0406), and it could also be seen that the OS of the control group (1-year OS rate 100%) had a better trend than HFA group (1-year OS rate 50%). In the B-ALL patients, the OS of the control group (1-year OS rate 100%) was higher than HFA group (1-year OS rate 45%, P=0.0189), although there was no significant difference in CR rate. High-grade CRS (≥3) occurred in 56.25% of the patients, and the mortality rate was 56.25%, which was significantly higher than control group (12.5% and 12.5%, P=0.009). The peak serum ferritin level in the patients who died of CRS was significantly higher than others (P=0.0168). Regardless of whether the CAR T-related MAS diagnostic criteria were met, there was no significant difference in ORR and OS in HFA group, however patients with MAS showed a higher rate of high-grade CRS. Interestingly, in our study, glucocorticoid intervention in HFA group showed little impact on expansion of CAR-T cells, whether compared with control group or compared within HFA group by dividing patients into high and low dosage subgroups based on the median dose of glucocorticoid. High mortality was observed in patients with CAR T-cell-related HFA. Early glucocorticoid intervention might be worth trying to improve the safety of CAR T therapy in these patients.

## Introduction

Immunotherapy using genetically engineered T-cells to express a chimeric antigen receptor (CAR) is rapidly emerging as a promising new treatment for hematological malignancies. Treatment with chimeric antigen receptor-engineered (CAR) T-cells has demonstrated higher complete remission (CR) rates and improved survival compared with chemotherapy for relapsed/refractory B-cell malignancies (1–3). However, CAR T-cell therapy is associated with unique acute toxicities, which can be severe or even fatal, such as cytokine release syndrome (CRS) and immune effector cell-associated neurotoxicity syndrome (ICANS), which occur as a result of T-cell overactivation (1, 4).

During CAR T-cell therapy, macrophage-activation syndrome (MAS), characterized by severe immune activation, lymph histiocytic infiltration, and immune response-mediated multiorgan failure, can occur (5). A total of 32.8% of ALL patients who received CD22-targeted CAR T-cell therapy experienced hemophagocytic lymphohistiocytosis (HLH)/MAS (6). However, in another multicenter retrospective study, the rate of MAS following CAR T-cell therapy was only 3.48% (7/201) (7). Fulminant and refractory HLH/MAS was observed in ~1% of all patients treated with CAR T-cell therapy, and special therapy, anti-IL-6 therapy, and corticosteroid treatment are necessary in addition to supportive care (4). If not treated promptly, refractory HLH/MAS is associated with high mortality (8). However, the diagnosis of MAS is difficult in the context of CRS, and many diagnostic criteria of MAS are not specific, such as fever and pancytopenia.

Monitoring ferritin levels can be helpful in the diagnosis of CAR T-cell-related MAS, and it is an essential condition of diagnosis that patients must have peak ferritin levels of >10,000 ng/ml during the CRS phase (4, 9). CAR T-cell-activated macrophages that contribute to highly elevated ferritin also function as antitumor agents (10, 11). Furthermore, higher serum ferritin levels may be a possible indication of a prolonged ICU hospitalization of patients treated with axi-cel (CAR19) and may be significantly associated with a higher grade of neurotoxicity (12, 13). However, the prognosis of patients with CAR T-cell-related HFA is unclear.

At our center, sequential infusion of CAR19/22 T cells was efficacious and reduced the rate of antigen-escape relapse in B-cell malignancies (14). However, the clinical features and outcomes of patients with CAR T-cell-related HFA (above 10,000 ng/ml) remain unclear. This study focused on the incidence, clinical characteristics, and therapeutic strategy of CAR T-cell-related ferritin levels above 10,000 ng/ml and these patient’s response to CAR T therapy.

## Materials and methods

### Patient characteristics

From January 17, 2018, to April 30, 2020, 129 patients with refractory/relapsed (r/r) B-cell non-Hodgkin’s lymphoma (B-NHL) and 56 patients with r/r acute B lymphoblastic leukemia (B-ALL) were enrolled in a clinical trial of sequential infusion of CAR19/22 T-cell therapy (Registry number ChiCTR-OPN-16008526 at http://www.chictr.org.cn). Among them, 8 lymphoma patients (8/129, 6.2%) and 8 leukemia patients (8/56, 14.3%), because of CRS, had high levels of ferritin (greater than 10,000 ng/ml [normal range, 30-400 ng/ml]). In this study, ferritin levels greater than 10,000 ng/ml were defined as HFA. Meanwhile, 16 cases with the lowest peak values of ferritin were grouped as controls, including 8 B-NHL and 8 B-ALL patients. This study was approved by Tongji Hospital, Tongji Medical College, Huazhong University of Science and Technology, in compliance with the Declaration of Helsinki. For details of this trial, refer to our previous study (14).

All patients were diagnosed based on the World Health Organization classification for hematological malignancies. Tumor burden was assessed by bone marrow examination, B ultrasound, positron emission tomography/computed tomography (PET/CT) or CT. Bulky disease was defined as a tumor mass 7.5 cm in maximal diameter (15). Ann Arbor stage and the International Prognostic Index (IPI) were evaluated for lymphoma patients. Next-generation sequencing (NGS), karyotype analysis, fusion gene detection, and fluorescence *in situ* hybridization (FISH) were performed for molecular genetic evaluation. In B-ALL, a complex karyotype was defined as ≥5 chromosome abnormalities (16). The date of the last follow-up was June 20, 2021.

### Generation of CAR T-cells and CAR T regimen

T cells were obtained from the patients. The lentiviral vector encoded anti-CD19 or anti-CD22 single-chain variable fragments, CD28, 4-1BB costimulatory domains and CD3-z signaling domains. Transfection, expansion and examination for effectiveness *in vitro* were implemented before infusion. The details were described in our previous study (14). An FC regimen (fludarabine 25 mg/m^2^ and cyclophosphamide 20 mg/kg daily for 3 days) was administered for lymphodepletion before CAR T-cell therapy. Anti-CD22 CAR T-cells were intravenously infused, followed by an infusion of anti-CD19 CAR T-cells the next day.

### Response assessment and monitoring

The treatment response was assessed after infusion according to the Lugano response criteria (17). The severity of cytokine release syndrome (CRS) and CAR T-cell-related encephalopathy syndrome (ICANS) was graded based on the CAR T-cell-therapy-associated TOXicity (CARTOX) Working Group Consensus (4) and the Common Terminology Criteria for Adverse Events (CTCAE) version 5.0 (18). The diagnostic criteria of CAR T-cell-related MAS are based on the recommendations of the CARTOX Working Group (4) and meet three of the following conditions: 1) The peak time of ferritin occurred during the CRS stage. 2) There was no obvious infection when a fever was present. 3) There was no evidence of primary disease progression during the peak of ferritin.

### Detection of CAR copy number in the peripheral blood

CAR gene copies were detected by droplet digital polymerase chain reaction (ddPCR) (19).

### Statistical analysis

Statistical analysis was performed with SPSS 18.0 software (IBM, Armonk, NY, USA). Continuous data are described as medians with ranges, while categorical data are described as frequencies (percentages). The independent t-test was used to compare continuous data between two groups. In contrast, the chi-square test or Fisher’s exact test was used to compare categorical data. GraphPad Prism 8.0 software (GraphPad Software Inc., San Diego, CA, USA) was used to plot the diagrams. Survival was analyzed with the log-rank test. P < 0.05 was considered statistically significant.

## Results

### Clinical characteristics

The clinical baseline characteristics of the B-NHL and B-ALL cohorts (each cohort included 8 patients with HFA and 8 control cases) are shown in **Tables 1**, **2**.

**Table 1:** Clinical characteristics of B-cell lymphoma patients.

	HFA (N = 8)	No HFA (N = 8)	*P*
**Overall Characteristics**			
Median age (range)——yr	50 (34-63)	41.5 (27-66)	*0.3074*
Male sex ——no. (%)	4 (50.0)	6 (75.0)	*0.6084*
**Histological subtype——no. (%)**			
DLBCL, NOS^#^	3 (37.5)	5 (62.5)	*0.6193*
DLBCL transformed from follicular lymphoma	1 (12.5)	1 (12.5)	>*0.9999*
DLBCL transformed from marginal zone lymphoma	0 (0.0)	1 (12.5)	>*0.9999*
High-grade B-cell lymphoma	2 (25.0)	1 (12.5)	>*0.9999*
Mantle cell lymphoma	1 (12.5)	0 (0.0)	>*0.9999*
Marginal zone lymphoma	1 (12.5)	0 (0.0)	>*0.9999*
**Ann Arbor stage——no. (%)**			
Stage III	0 (0.0)	2 (25.0)	*0.4667*
Stage IV	8 (100.0)	6 (75.0)	
**IPI——no. (%)**			
1-2	0 (50.0)	4 (87.5)	*0.0769*
3-4	8 (50.0)	4 (12.5)	
**Genetic abnormalities——no. (%)**			
Double-/triple-hit rearrangement	2 (25.0)	1 (12.5)	>*0.9999*
TP53 deletion/mutation	3 (37.5)	4 (50.0)	>*0.9999*
**Disease burden before treatment——no. (%)**			
Bulky ≥ 7.5 cm	4 (50.0)	1 (12.5)	*0.2821*
With extranodal involvement	7 (37.5)	4 (12.5)	*0.2821*
With ≥2 sites of extranodal involvement	5 (25.0)	3 (12.5)	*0.6193*
**Previous therapies**			
Median (range) lines	3 (2-4)	3 (2-4)	*0.2851*
≥3 prior lines of therapy——no. (%)	7 (87.5)	6 (75.0)	>*0.9999*
≥4 prior lines of therapy——no. (%)	3 (37.5)	1 (12.5)	*0.5692*

^#^diffuse large B-cell lymphoma, not otherwise specified.

*Central nervous system.

**Table 2:** Clinical characteristics of ccute lymphoblastic leukemia patients.

	HFA (N = 8)	No HFA (N = 8)	*P*
**Overall Characteristics**			
Median age (range)——yr	34 (22-50)	34 (15-58)	*0.8242*
Male sex——no. (%)	3 (37.5)	1 (12.5)	*0.2821*
**Cytogenetic features——no. (%)**			
Philadelphia chromosome-positive	3 (37.5)	5 (62.5)	*0.6193*
Philadelphia chromosome-like (Ikaros 6)	1 (12.5)	0 (0.0)	>*0.9999*
Complex karyotype	4 (50.0)	1 (12.5)	*0.2821*
E2A/PBX1 fusion gene	1 (12.5)	0 (0.0)	>*0.9999*
**Disease burden before treatment**			
Median bone marrow blasts (range)	64.0% (6-99.5%)	17.5% (0^#^-93.9%)	*0.1723*
CNS* involvement——no. (%)	4 (50.0)	1 (12.5)	*0.2821*
**Previous therapies**			
Median (range) lines	2 (2-5)	2 (2-5)	*0.8178*
≥3 prior lines of therapy——no. (%)	2 (25.0)	1 (12.5)	>*0.9999*
**Previous allogeneic stem-cell transplantation——no. (%)**	1 (12.5)	1 (12.5)	>*0.9999*

^#^the patient with 0% bone marrow blasts had an orbital mass and CNS involvement.

*Central nervous system.

The median age was 50 years (34-63 years) and 41.5 years (27-66 years) for the HFA and control cohorts of B-NHL patients, respectively, while the sex ratio (male/female) was 1.00 and 3.00. Among the 8 B-NHL patients with HFA, 3 had diffuse large B-cell lymphoma not otherwise specified (DLBCL NOS), 1 had DLBCL transformed from follicular lymphoma (FL), 2 had high-grade B-NHL (HGBL), 1 had FL, and 1 had marginal zone lymphoma (MZL). For the B-NHL control group, there were 5 DLBCL NOS, 1 DLBCL transformed from FL, 1 DLBCL transformed from MZL and 1 HGBL. All HFA patients (8/8, 100%) and 6 control cases (6/8, 75%) were in the advanced stage (Ann Arbor stage IV). All patients in the HFA group had an IPI score of 3 or more, while in the control group, half of the patients had an IPI score of 1-2. Five out of 8 HFA patients (62.5%) had high-risk molecular abnormalities, including double-/triple-hit rearrangements (2/8, 25.0%) and TP53 deletion/mutation (3/8, 37.5%). In the control group, the number was 5 (62.5%) with 1 double-/triple-hit rearrangement (12.5%) and 4 TP53 deletions/mutations (50.0%). Bulky disease was seen in 4 HFA patients (4/8, 50.0%) and in 1 case (12.5%) in the control group. Extranodal lesions, including bone marrow (3/8, 37.5%), central nervous system (CNS) (2/8, 25.0%), liver (2/8, 25.0%), spleen (1/8, 12.5%), lung (1/8, 12.5%) and gastrointestinal tract (2/8, 25.0%), were observed in most HFA patients (7/8, 87.5%), and 5 of the patients had two or more extranodal sites. In the control group, 3 out of 4 extranodal involved cases had 2 sites of extranodal lesions. All 16 B-NHL patients had received a median of 3 lines of prior treatment, and 3 (3/8, 37.5%) and 1 (1/8, 12.5%) in the HFA and control group underwent fourth-line treatment, respectively.

Among the 16 B-ALL patients, the median age was 34 years (22-50 years) and 34 years (15-58 years) for the HFA and control groups, with sex ratios of 0.60 and 0.125, respectively. All HFA B-ALL patients had high-risk molecular abnormalities, including BCR-ABL (3/8, 37.5%; one of the patients had T315I mutation), ph-like (Ikaros 6) (1/8, 12.5%), E2A/PBX1 fusion gene (1/8, 12.5%) and complex karyotypes (4/8, 50.0%). In the control group, 5 patients (62.5%) had high-risk molecular abnormalities with all 5 BCR-ABL (62.5%), and two of them also had T315I mutations and complex karyotypes. The number of bone marrow blasts in the 8 HFA patients and 8 control cases was 64.0% (6.0% to 99.5%) and 17.5% (0% to 93.9%; the patient with 0% bone marrow blast had an orbital mass and CNS involvement), respectively. Four of 8 HFA patients (50.0%) and 1 of 8 control cases (12.5%) developed CNS infiltration. Patients in both groups had a median prior therapy of 2 lines (range, 2 to 5 lines). Two HFA patients (2/8, 25.0%) and 1 control case (1/8, 12.5%) received at least third-line treatments. One patient in the HFA group and control group previously received allogeneic hematopoietic stem-cell transplantation (allo-HSCT).

The small sample size of this study may be a possible limitation of detecting significant differences between the baseline characteristics of the two groups. However, a tendency could still be observed that compared with the control group, B-NHL patients with HFA had a higher proportion of cases with 3-4 IPI scores (P=0.0769), bulky disease (P=0.2821) and extranodal lesions (P=0.2821), while B-ALL patients with HFA had higher bone marrow blasts (P=0.1723) and more CNS-involved cases (P=0.2821).

### Outcome

**Figure 1:**
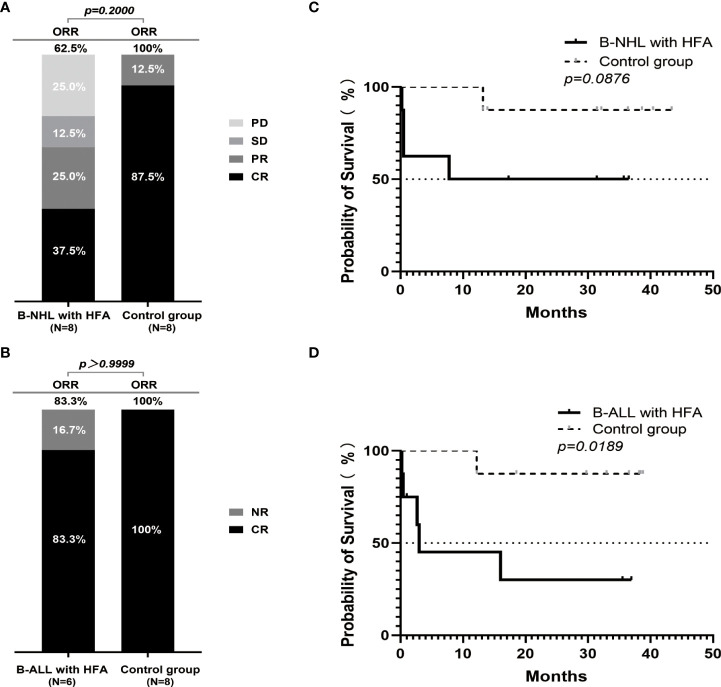
Outcomes of r/r B-NHL and r/r B-ALL cases according to hyperferritinaemia status after the CAR19/22 T-cell therapy. The percentage of patients with CR, PR, SD, PD or CR, NR as the best response in 16 patients with r/r B-NHL (8 in HFA group and 8 in control group) **(A)** and 14 evaluable patients with r/r B-ALL (6 in HFA group and 8 in control group) **(B)**. Overall survival in the B-NHL **(C)** and B-ALL **(D)** cohorts on the basis of hyperferritinaemia status (each cohort included 8 patients with HFA and 8 control cases).

Among the 8 B-NHL patients with HFA, the objective response rate (ORR) was 62.5%, including 3 with CR (3/8, 37.5%), 2 with partial remission (PR) (2/8, 25%), 1 with stable disease (SD) (1/8, 12.5%) and 2 with progressive disease (PD) (2/8, 25%). In contrast, among the 8 B-NHL patients without HFA, the ORR was 100%, including 7 with CR (7/8, 87.5%) and 1 with partial remission (PR) (1/8, 12.5%) (**Figure 1A**). The CR rate of patients with HFA was significantly lower than that of patients in the control group (P=0.0406). Among the 8 B-NHL patients with HFA, 3 CR patients achieved a sustained remission. One PR patient received HSCT therapy followed by CAR T therapy and achieved a CR, and the other PR patient’s disease progressed again. In contrast, most patients in the control group (7/8, 87.5%) were still alive, and only one patient died of disease progression (**Supplementary Figure 1A**).

Among the 8 B-ALL patients with HFA, 2 patients died of severe CRS after CAR T-cell infusion before evaluation, 5 of 6 evaluable patients achieved a CR (5/6 83.3%), and only 1 patient had no response. In the control group, all of the patients achieved a CR (**Figure 1B**). Among the 5 B-ALL patients with HFA who achieved a CR, one was lost to follow-up in the second month after CAR T-cell infusion, and the other 4 underwent three- to thirty-seven-months of follow-up. Three of them received allo-HSCT, one achieved a sustained CR, one relapsed and the last one died of severe infection during transplantation. Conversely, in the control group, only one patient died of severe infection after transplantation, and the others were all alive (**Supplementary Figure 1B**).

In the B-ALL patients, the OS of the patients without HFA was significantly higher than that of the patients with HFA (P=0.0189). In patients with B-NHL, although there was no significant difference in OS between the patients with and without HFA (P=0.0876), it could also be seen that the OS of the patients without HFA had a better trend than that of the patients with HFA (**Figures 1C, D**). Meanwhile, B-NHL patients with HFA and bulky disease had a significantly worse OS than those without bulky disease (P=0.0069) (**Supplementary Figure 1C**). There was no difference in OS between the B-NHL patients with or without high-risk molecular abnormalities (P=0.0511) (**Supplementary Figure 1D**). However, the proportion of patients with high-risk molecular abnormalities among those with bulky disease was higher than in those with non-bulky disease (P=0.0240) (**Supplementary Figure 1E**).

### Safety

**Figure 2:**
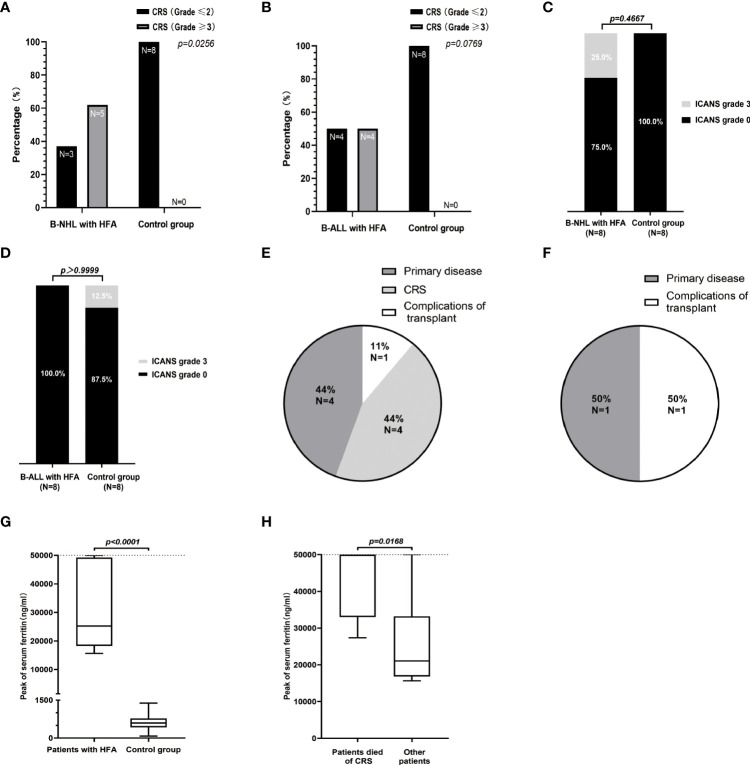
Safety assessments of the 32 patients in B-NHL and B-ALL cohorts. The percentage of patients with high-grade (≥3) and low-grade (≤2) CRS in the B-NHL **(A)** and B-ALL **(B)** cohorts on the basis of hyperferritinaemia status. **(C, D)** ICANS developed in 3 patients (including 2 B-NHL patients in HFA group and 1 B-ALL patient in control group) and all were of grade 3. Causes of death for 9 cases of HFA group **(E)** and 2 cases of control group **(F)**. **(G)** The medians of the peak values of serum ferritin in HFA group (n=16) and control group (n=16) were 25257 (range, 15650-50000) ng/ml and 593.1 (range, 77.6-1384) ng/ml, respectively. **(H)** In the 16 HFA patients, the medians of the peak values of serum ferritin in patients who died of CRS (n=4) and other patients (n=12) were 50000 (range, 27370-50000) ng/ml and 21049.5 (range, 15650-50000) ng/ml, respectively. The horizontal lines in each box represent the median values, and the lower and upper boundaries of each box represent the 25th and the 75th percentiles, respectively, and the whiskers represent the minimum and maximum range. The detection upper limit of serum ferritin was 50000 ng/ml.

All 16 HFA cases developed CRS within 14 days after CAR T-cell infusion; among them, 7 cases (7/16, 43.8%) were grade 1~2, 5 cases (5/16, 31.3%) were grade 3~4 and 4 cases (4/16, 25.0%) were grade 5. Two patients with lymphoma developed ICANS of grade 3~4. Compared with the control group, patients with HFA had a significantly higher high-grade CRS (≥3) rate in B-NHL (P=0.0256) (**Figure 2A**). However, no significant difference was found for B-ALL patients or in the distribution of ICANS grades between patients with and without HFA for both lymphoma and leukemia (**Figures 2B-D**). Nine of 16 patients with HFA (56.25%) died (4 died of CAR T-related MAS, 4 died of tumor progression and 1 died of an infection after allo-HSCT) (**Figure 2E**). Only two of 16 patients without HFA (12.5%) died (1 died of tumor progression, and 1 died of an infection after allo-HSCT) (**Figure 2F**).

The levels of serum ferritin in both HFA group and control group were significantly increased during CRS (**Supplementary Figures 2A, B**). The levels of serum ferritin and IL-6 in the 16 patients with HFA peaked at a median of 8 days (range 5 to 13 days) and 7 days (range, 3-15 days) after infusion, with median values of 25256.5 ng/ml (normal value 30-400 ng/ml) and 2158.5 pg/ml (normal value < 7 pg/ml), respectively (**Supplementary Figures 2C, D**). The peak serum ferritin levels in patients with HFA were significantly higher than those in the patients without HFA (P<0.0001), furthermore, among the 16 patients with HFA, the peak serum ferritin levels in the patients who died of CRS were significantly higher than those in the other patients (P=0.0168)(**Figures 2G, H**).

The MAS-related clinical characteristics of the 16 patients with HFA are shown in **Table 3**. Among them, 6 lymphoma patients (6/8, 75.0%) and 3 leukemia patients (3/8, 37.5%) met the MAS diagnostic criteria. Thus, the rates of CAR T-cell-related MAS in our center from January 17, 2018, to April 30, 2020, were 4.7% (6/129) in patients with lymphoma and 5.4% (3/56) in patients with leukemia. Seven lymphoma cases (7/8, 87.5%) and 4 leukemia cases (4/8, 50.0%) had ≥ 3-grade liver toxicities. Six lymphoma cases (6/8, 75.0%) and 3 leukemia cases (3/8, 37.5%) had ≥ 3-grade pulmonary edema. In the control group, lymphoma patients, but not leukemia patients, had significantly fewer and less severe lung and liver symptoms than the HFA group (**Figures 2E-H**). None of the 32 patients had ≥ 3-grade kidney toxicity. Triglycerides were detected in 5 of the 16 patients with HFA; among them, 3 of 4 lymphoma patients had a high serum triglyceride level (> 3 mmol/L), and 1 leukemia patient who had serum triglyceride tests was normal. All of them had high peak values of soluble IL-2 receptor, which were all higher than the upper detection limit (7500 u/ml, normal range 223-710 u/ml). Hypofibrinogenemia (< 1.5 g/L) was found in 87.5% (7/8) of lymphoma patients and 62.5% (5/8) of leukemia patients, and the minimums of fibrinogen after the CAR-T cells infusion of the HFA patients were significantly lower than those in the control group (P=0.0021 for B-NHL cohort and P=0.0162 for B-ALL cohort) (**Supplementary Figures 2I, J**). For the bone marrow tests, these 16 patients with HFA were critically ill during the CRS phase, and considering that the results could hardly affect the treatment strategy, bone marrow biopsy was not performed.

**Table 3:** Manifestations of hyperferritinaemia, treatment approach and outcome.

Pt	Type of disease	Mas	Pertinent laboratory results				CRS	ICANS	Treatment				Outcome
			Baseline value of ferritin	Peak values	Lowest value of FIB		Grade ≥ 3 pulmonary edema		Steroid utilization (Total dose)	Tocilizumab utilization	Plasma-pheresis	Maximum respiratory support	
1	DLBCL	Y	896	AST = 192 Ferritin = 16479 sIL-2R > 7500	0.88	Y	Grade 3	No	333 mg	Y	Y	High flow oxygen (5 L/min)	Full resolution with treatment
2	MZL	Y	2337.6	ALT = 190 Ferritin = 24185 sIL-2R > 7500	1.03	Y	Grade 4	Grade 3	573 mg	N	Y	BIPAP	Full resolution with treatment
3	HBCL	Y	981	AST = 390 T.Bili = 352.13 Ferritin = 27370 sIL-2R > 7500	0.82	Y	Grade 5	No	213 mg	Y	Y	High flow oxygen (7 L/min)	Died of complications of CRS
4	HBCL	N	4943.9	Ferritin = 19082 sIL-2R > 7500	1.57	N	Grade 2	No	40 mg	Y	Y	Medium flow oxygen (3 L/min)	Died of tumor progression
5	DLBCL	Y	2742.6	AST = 1670 T.Bili = 147.4 Ferritin > 50000 sIL-2R > 7500	1.04	Y	Grade 5	No	2153 mg	Y	Y	Invasive mechanical ventilation	Died of complications of CRS
6	MCL	N	1599	AST = 168 Ferritin > 50000 sIL-2R > 7500	1.29	N	Grade 2	No	160 mg	N	Y	Medium flow oxygen (3 L/min)	Full resolution with treatment (Proceeded to ibrutinib)
7	DLBCL	Y	1611	ALT = 277 AST = 393 T.Bili = 89.93 Ferritin = 26328 sIL-2R > 7500	0.94	Y	Grade 3	No	400 mg	N	Y	High flow oxygen (5 L/min)	Full resolution with treatment (died of tumor progression)
8	DLBCL	Y	1600	AST = 180 T.Bili = 28.5 Ferritin = 20349 sIL-2R > 7500	0.93	Y	Grade 2	Grade 3	733 mg	Y	Y	High flow oxygen (5 L/min)	Full resolution with treatment (Proceeded to auto-HSCT)
9	B-ALL	Y	1390	ALT = 431 AST = 782 T.Bili = 196.6 Ferritin > 50000 sIL-2R > 7500	1.87	Y	Grade 5	No	4447 mg	N	Y	Invasive mechanical ventilation	Died of complications of CRS
10	B-ALL	Y	1410	AST = 466 Ferritin > 50000 sIL-2R > 7500	0.68	Y	Grade 5	No	400 mg	N	Y	BIPAP	Died of complications of CRS
11	B-ALL	N	2789	Ferritin = 16147 sIL-2R > 7500	0.98	N	Grade 2	No	146 mg	N	Y	Low flow oxygen (2 L/min)	Full resolution with treatment (Proceeded to HSCT and died of complications of Allo-HSCT)
12	Ph-like B-ALL	Y	392.8	ALT = 135 Ferritin = 18000 sIL-2R > 7500	1.09	Y	Grade 3	No	307 mg	N	Y	High flow oxygen (6 L/min)	Full resolution with treatment (Died of tumor progression)
13	Ph+ ALL	N	360.3	Ferritin = 21750 sIL-2R > 7500	0.83	N	Grade 2	No	167 mg	N	Y	Without oxygen	Full resolution with treatment
14	Ph+ ALL	N	4244	T.Bili = 84.18 Ferritin = 35488 sIL-2R > 7500	2.7	N	Grade 3	No	80 mg	N	N	Low flow oxygen (2 L/min)	Full resolution with treatment (Proceeded to dasatinib)
15	B-ALL	N	2193.9	T.Bili = 74.98 Ferritin = 46919 sIL-2R > 7500	0.94	N	Grade 2	No	916 mg	N	Y	Without oxygen	Full resolution with treatment (Died of tumor progression)
16	Ph+ ALL	N	2358	Ferritin = 15650 sIL-2R > 7500	2.23	N	Grade 2	No	40 mg	N	Y	Low flow oxygen (2 L/min)	Full resolution with treatment

MAS, macrophage activation syndrome; DLBCL, diffuse B-cell lymphoma; MZL, marginal zone lymphoma; HBCL, high-grade B-cell lymphoma; MCL, mantle cell lymphoma; B-ALL, B-cell acute lymphoblastic leukemia; Ph+ALL, Philadelphia chromosome positive acute lymphoblastic leukemia; Ph-like ALL, Philadelphia chromosome-like acute lymphoblastic leukemia; Y, yes; N, no; ALT, alanine aminotransferase (U/L); AST, aspartate aminotransferase (U/L), Ferritin reported as ng/ml; T.Bili, total bilirubin (μmol/L); sIL-2R, soluble IL-2 receptor (u/ml); FIB, fibrinogen (g/L); CRS, cytokine-release syndrome; ICANS, immune effector cell-associated neurotoxicity syndrome; No, did not occur.

**Figure 3:**
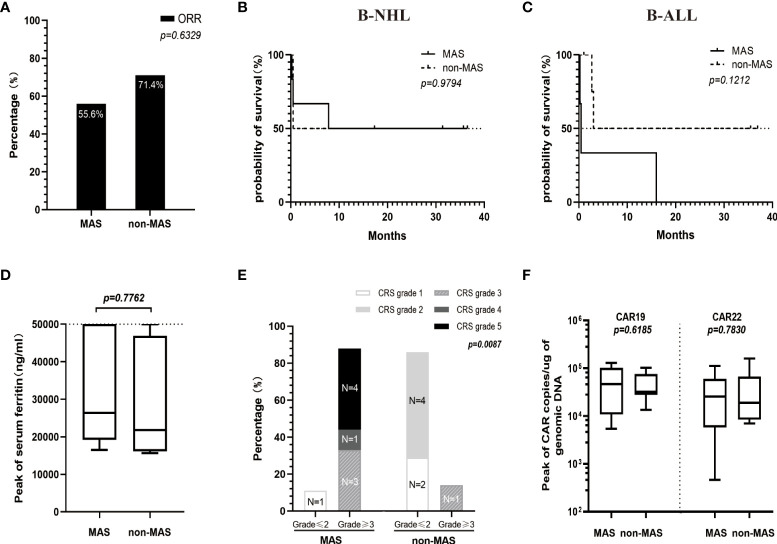
Comparison between patients of the HFA group with MAS (n=9) and without MAS (n=7) in ORR **(A)**, OS **(B, C)**, levels of serum ferritin **(D)**, CRS grade **(E)** and the peaks of CAR transgene copies **(F)**. **(D)** The medians of the peak values of serum ferritin in patients with or without MAS were 26328 (range, 16479-50000) ng/ml and 21750 (range, 15650-50000) ng/ml, respectively. **(E)** Patients with MAS had a significantly higher high-grade CRS (≥3) rate than patients without MAS (P=0.0087). **(F)** The peak values for CAR19 and CAR22 transgene copies in the MAS group (median 46393, range 5441-129375 copies/µg for CAR19 and median 25688, range 464-112033 copies/µg for CAR22) were not significantly different from those in the non-MAS group (median 31909, range 13473-102707 copies/µg for CAR19 and median 18924, range 6970-159171 copies/µg for CAR22).

Among the 16 patients with HFA, there was no difference in OS, ORR, levels of serum ferritin or peaks of CAR transgene copies between patients with and without CAR T-cell-related MAS. However, patients with MAS had a more serious CRS than those without MAS (P=0.0087) (**Figures 3A-F**).

### Treatment and CAR T-cell kinetics

**Figure 4:**
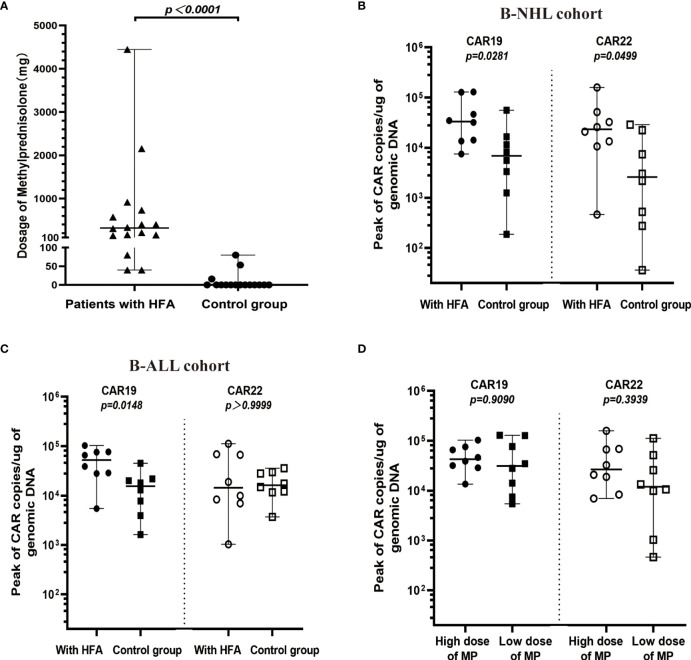
Cellular kinetics of CAR transgenes in peripheral blood. **(A)** Comparison between HFA group (n=16) and control group (n=16) in dosages of MP (median 320 mg, range 40-4447 mg, and median 0 mg, range 0-80 mg, respectively), only 3 patients in the control group received MP after the CAR-T cell infusion. **(B)** In B-NHL cohort, the peak values for CAR19 and CAR22 transgene copies in the HFA group (median 33222, range 7458-129375 copies/µg for CAR19 and median 23284, range 464-159171 copies/µg for CAR22) were all significantly higher than those in the control group (median 6864, range 187-55774 copies/µg for CAR19 and median 2588, range 36-28740 copies/µg for CAR22). **(C)** In B-ALL cohort, the peak values for CAR19 (median 52291, range 5441-102707 copies/µg), but not for CAR22 (median 14433, range 1033-112033 copies/µg) transgene copies in the HFA group were significantly higher than those in the control group (median 15519, range 1615-45078 copies/µg for CAR19 and median 16170, range 3703-35729 copies/µg for CAR22). **(D)** According to the median dose of MP (320 mg), 16 patients with HFA were divided into a low-dose MP group and a high-dose MP group. The peaks of CAR19 and CAR22 transgene copies in the high-dose MP group (median 31679, range 5441-129375 copies/µg for CAR19 and median 11949, range 464-112033 copies/µg for CAR22) were not significantly different from those in the low-dose MP group (median 42697, range 4770-102707 copies/µg for CAR19 and median 26669, range 6970-159171 copies/µg for CAR22).

Methylprednisolone (MP) was administered for CRS in all 16 patients with HFA, and the median of the initial application time was 6 days (range, 3-12 days) after infusion. The median MP dosages were 1276.5 mg (range, 213-4447 mg) for 4 patients who died of serious CRS, 307 mg (range, 146 to 916 mg) for 5 patients who died of other reasons, and 167 mg (range, 40 to 733 mg) for 7 survivors. Notably, the MP dosages in 4 patients with HFA who died of serious CRS were higher than those in the other 12 HFA cases (median 237 mg, range 40-916 mg) (P=0.0173). Only 3 of 16 patients in the control group received MP for CRS (median 53.33 mg, range 16-80 mg). Unsurprisingly, the patients with HFA had significantly higher dosages of MP than those without HFA (P<0.0001) (**Figure 4A**). Moreover, most HFA patients also received plasmapheresis and hemofiltration, and some of them underwent tocilizumab therapy. Advanced life support, including vasopressor drug pumps, bipaps, invasive mechanical ventilation and oxygen therapy, was performed as needed (**Table 3**).

Both CAR19 and CAR22 T cells expanded well in all patients with HFA. During the 14 days after CAR T-cell infusion, the peaks for CAR19 and CAR22 in the peripheral blood were 36767 copies/µg (range, 4770-129375) and 23283.5 copies/µg (range, 464-159171), respectively. In the control group, the peaks for CAR19 and CAR22 in the peripheral blood were 11393 copies/µg (range, 1255-55774) and 12141 copies/µg (range, 275-159171), respectively (**Supplementary Figures 3A, B**). The peaks for CAR19 in all HFA patients and the peaks for CAR22 in HFA patients with B-NHL were significantly higher than those in the control group (**Figures 4B, C**). All patients with HFA were divided into two groups—a high dose and a low dose of methylprednisolone—based on its median dose of 320 mg. There was no significant difference in the peaks of CAR19 and CAR22 between the two groups (**Figure 4D**). The peaks of CAR19 (P=0.0905) and CAR22 (P=0.3349) in CR and PR patients were not higher than those in the nonresponding patients (**Supplementary Figure 3C**). The patients who died of CRS had similar CAR transgene copies to the other patients for CAR19 and CAR22 (**Supplementary Figure 3D**).

## Discussion

In our study, 16 out of 185 patients (129 with lymphoma and 56 with leukemia) had high serum ferritin levels (above 10,000 ng/ml) after CAR T-cell infusion. The incidence of CAR T-related MAS in 185 cases was 4.86% (9/185), and in 16 cases, the incidence of CAR T-related MAS was 56.25% (9/16). Although CAR T-cell-related HFA and MAS are rare adverse events, these adverse events often have very poor outcomes.

For 16 cases with HFA, the CR rates were 37.5% in lymphoma and 83.3% in leukemia, which were lower than those in our previous report (50% and 96%) (14). Poor CR rates may be related to an extremely high tumor burden. For the 8 lymphoma cases, 4 cases had bulky disease, bone marrow (62% BM blast) was involved in one case, one case had primary cutaneous DLBCL-leg type and the other 2 cases had CNS or spine involvement. Three lymphoma patients who had no response to CAR T-cell therapy all had bulky disease, which is similar to previous research, suggesting that a high tumor burden, especially bulky disease, was associated with an inadequate therapeutic response (20–22). Four out of 8 leukemia cases had extramedullary infiltration. The leukemia patient with an extramedullary mass was a nonresponding patient. Some previous reports also implied that leukemia with an extramedullary mass had a poor response to CAR T-cell therapy (23, 24). In terms of the efficacy in our study, patients with high-risk genetic abnormalities were more likely to have a high tumor burden. It was probably that high-risk genetic abnormalities are closely related to chemoresistance (25–27). Compared with the control group, HFA patients also had a higher proportion of IPI scores, large masses, and extranodal involvement; a lower CR rate; and a more serious CRS. Therefore, early delivery of CAR T-cells, especially before chemoresistant clones dominate in the tumor tissue and become bulky disease, may induce a better response in these high-risk cases.

Meanwhile, high tumor burden might bring high CAR T-cell therapy risk. In our study, a higher rate of high-grade CRS was observed in patients with CAR T-related MAS than in patients with CAR T-related HFA only and our previous report (14). 4 patients died of severe CRS, and they all had CAR T-related MAS. The mortality (44.4%, 4/9) in patients with CAR T-related MAS was very high. Both a poor response to CAR T-cell therapy and severe CRS caused a shorter median OS (3 months and not reached in leukemia and lymphoma, respectively) compared with patients without HFA, as in our previous report (31 months for leukemia and 18 months for lymphoma) and other trials (12.9-13.3 months for leukemia and 12-21.1 months for lymphoma) (3, 23, 28, 29). Notably, higher the peaks for CAR19 in all HFA patients than those in the control group indicate a high tumor burden could lead to a rapid expansion of CAR T-cells, which led to severe side effects. Meanwhile, previous studies have also shown that a high tumor burden was positively correlated with CAR T-cell expansion, and these CAR T-cells induced the activation of macrophages. There was more macrophage infiltration in tumor tissue when the patient had a higher tumor burden (30, 31). The severity of CRS after CAR T-cell therapy was closely associated with the expansion and overactivation of CAR T-cells and CAR T-related macrophages (32–34).

Although the HFA patients received a higher dose of glucocorticoids than the control group, the peaks for CAR19 in all HFA patients and the peaks for CAR22 in HFA patients with B-NHL were significantly higher than those in the control group, which might indicate that glucocorticoid intervention had little impact on the expansion of CAR T-cells. However, there are many factors, in addition to glucocorticoids, affect the expansion of CAR T-cells, including the general condition of the patients, tumor burden, and the quality of the CAR T-cells (35–37). Notably, the patients with HFA had similar baseline characteristics and received a high dose of glucocorticoids after CAR T-cell infusion. Glucocorticoids also showed little impact on the expansion of CAR T-cells which was no significant difference between a high dose and a low dose of methylprednisolone. The median time of glucocorticoid use was 6 days after CAR T-cell infusion when the grade of CRS reached 2. The ZUMA 1 cohorts 4 and 6 showed that the occurrence of high-grade CRS was decreased when glucocorticoids were administered early, during CRS grade 1 or preventively before CRS (38). Based on the safety and above data, early glucocorticoid intervention might be useful to improve the safety of CAR T-cell therapy.

In our study, high serum ferritin (≥ 10,000 ng/ml) after CAR T infusion, which was observed in patients with heavy/mass disease burden or high-risk genetic abnormalities, may indicate a poor response and high risk of severe CRS. Early delivery of CAR T-cells and early glucocorticoid intervention during CAR T-cell therapy might improve the outcome of these patients.

## Data availability statement

The raw data supporting the conclusions of this article will be made available by the authors, without undue reservation.

## Ethics statement

The studies involving human participants were reviewed and approved by the Medical Ethics Committee of the Tongji Hospital, Tongji Medical College, Huazhong University of Science and Technology (TJ-IRB20160310). The patients/participants provided their written informed consent to participate in this study. Written informed consent was obtained in accordance withthe Declaration of Helsinki. This study is registered at www.chictr.org.cn as ChiCTR-OPN-16008526.

## Author contributions

LZ, NY, and XZ conceived and coordinated the study, designed, performed, and analyzed the experiments, and wrote the paper. TL, HJ, LJ, DW, and BX carried out the data collection and data analysis and revised the paper. All authors contributed to the article and approved the submitted version.

## Funding

This work was supported by the National Natural Science Foundation of China (Grant No. 8217011455).

## Acknowledgments

The authors would like to thank all members of the study team for their clinical support.

## Conflict of interest

The authors declare that the research was conducted in the absence of any commercial or financial relationships that could be construed as a potential conflict of interest.

## Publisher’s note

All claims expressed in this article are solely those of the authors and do not necessarily represent those of their affiliated organizations, or those of the publisher, the editors and the reviewers. Any product that may be evaluated in this article, or claim that may be made by its manufacturer, is not guaranteed or endorsed by the publisher.
