# Supplementary material for: Clinical characteristics and prognosis of 16 relapsed/refractory B-cell malignancy patients with CAR T-cell-related hyperferritinaemia
Source: Front Oncol. 2022 Oct 12;12:912689. doi: 10.3389/fonc.2022.912689 (PMC9600326; doi:10.3389/fonc.2022.912689)
Supplement: Supplementary file 1 [file DataSheet_1.docx]

**Clinical characteristics and prognosis of 16 relapsed/refractory B-cell malignancies patients with CAR T-cell related hyperferritinemia**

Lanlan Zhou^a¶^, Nanzhou Yu^b¶^, Tongjuan Li^b^, Hongyan Ji^b^, Lijun Jiang^b^, Di Wang^b^, Bin Xu^b^ and Xiaoxi Zhou*^b^

*^a^Department of Hematology, the First Affiliated Hospital of Guangdong Pharmaceutical University, Guangdong, China; ^b^ Department of Hematology, Tongji Hospital TongJi Medical College Huazhong University of Science & Technology, Wuhan, Hubei,430030, P. R. China.*

*^¶^These authors contributed equally to this work.*

****Corresponding author:*** *Xiaoxi Zhou, M.D, PhD.* *Tel: 86-27-83665007. E-mail: cello316@163.com.*

**Supplementary Data**

**Supplementary Figure 1**

**Supplementary Figure 2**

**Supplementary Figure 3**

**Supplementary Figure 1**


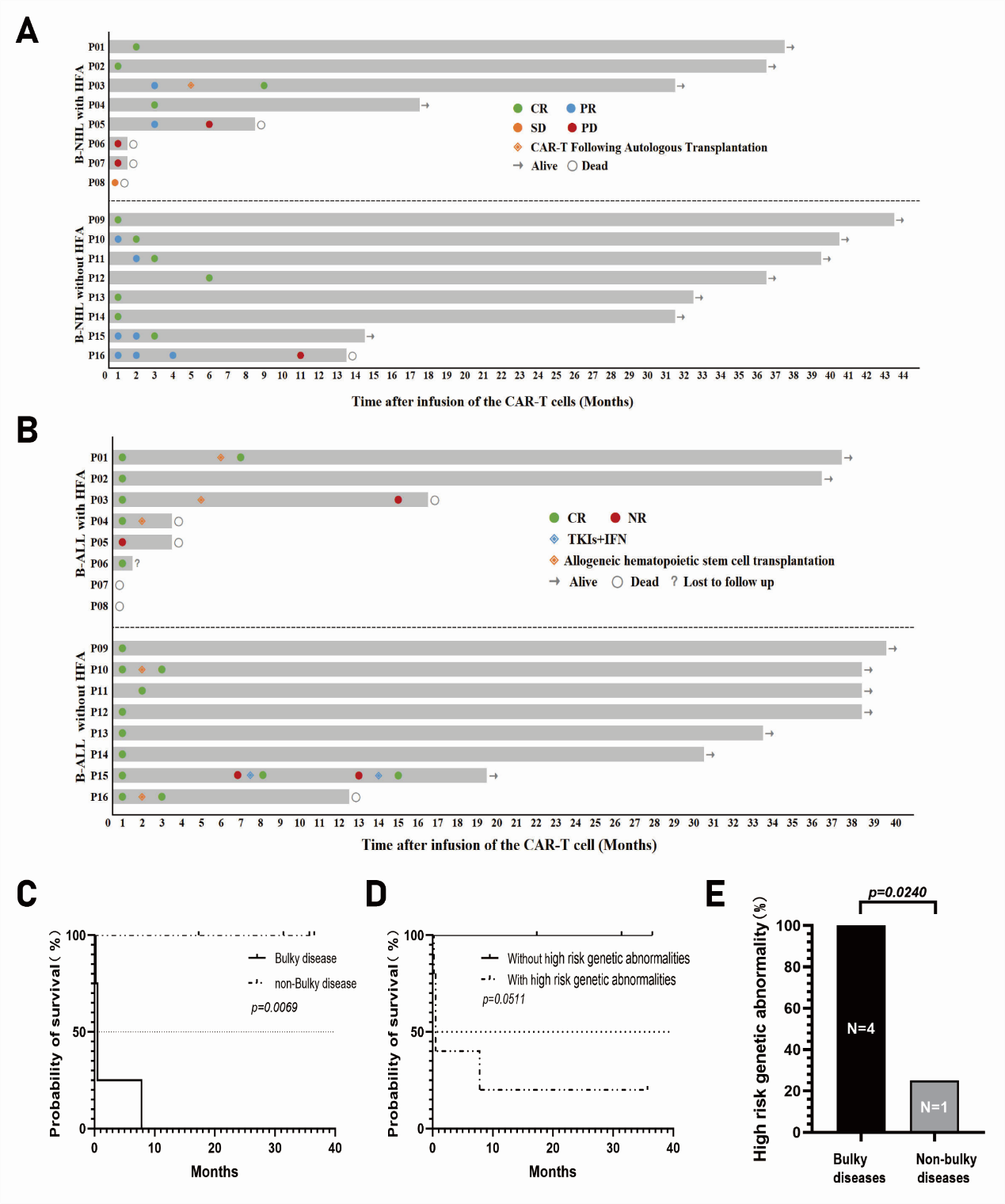


**S Figure.1.** Therapeutic effect and disease status of the 32 patients in B-NHL **(A)** and B-ALL **(B)** cohorts after the CAR19/22 T-cell therapy. Different colored dot represents the disease status and prismatic shows follow-up treatment. Arrows indicate the ongoing status until the expiry date of follow-up, and circles indicate the dead cases, and the question mark indicates a patient lost to follow-up. **(C)** The OS of patients in B-NHL with bulky disease was worse than non-bulky disease (P=0.0069). **(D)** There was no difference in OS between B-NHL patients with and without high-risk molecular abnormalities (double-/triple-hit rearrangements and the TP53 deletion/mutation) (P=0.0511). **(E)** B-NHL patients with bulky disease (n=4) had more rate of high-risk molecular abnormalities than without bulky disease (P=0.0240, n=4).

**Supplementary Figure 2**


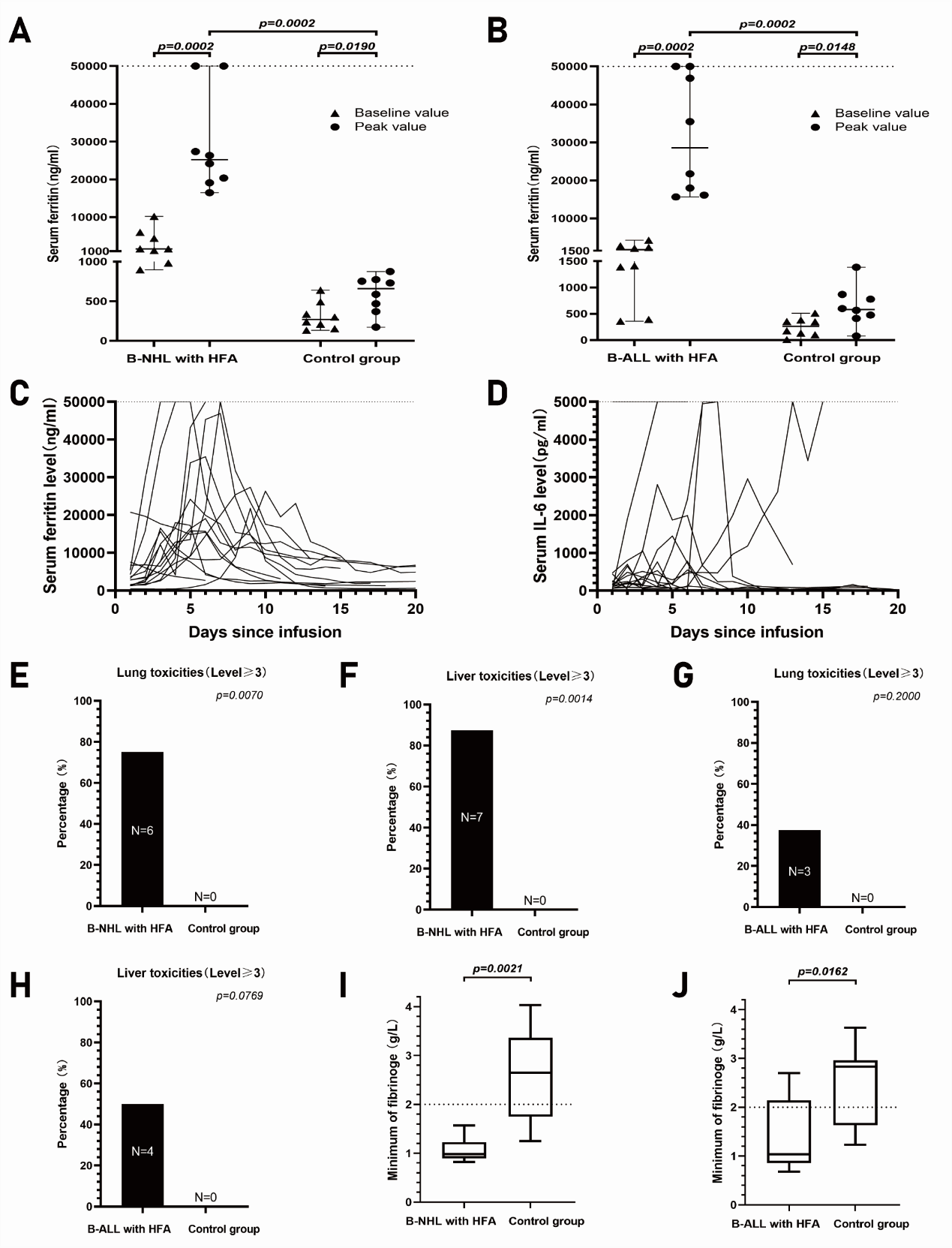


**S Figure 2.** In B-NHL **(A)** and B-ALL **(B)** cohorts, the levels of serum ferritin of both HFA group and control group were significantly increased during CRS compared to the baseline, and the peak serum ferritin levels in the HFA group were significantly higher than those in control group. **(C, D)** Levels of serum ferritin and IL-6 in all 16 patients of HFA group during the 20 days after CAR-T cell infusion. The upper limits of serum ferritin and IL-6 detected were 50000 ng/ml and 5000 pg/ml, respectively. **(E-H)** Comparison of severe lung and liver toxicities (level≥3) between the HFA group and the control group in B-NHL and B-ALL cohorts. **(I, J)** Comparison of the minimums of fibrinogen after the CAR-T cell infusion of the HFA group and those of the control group (P=0.0021 for B-NHL cohort and P=0.0162 for B-ALL cohort).

**Supplementary Figure 3**


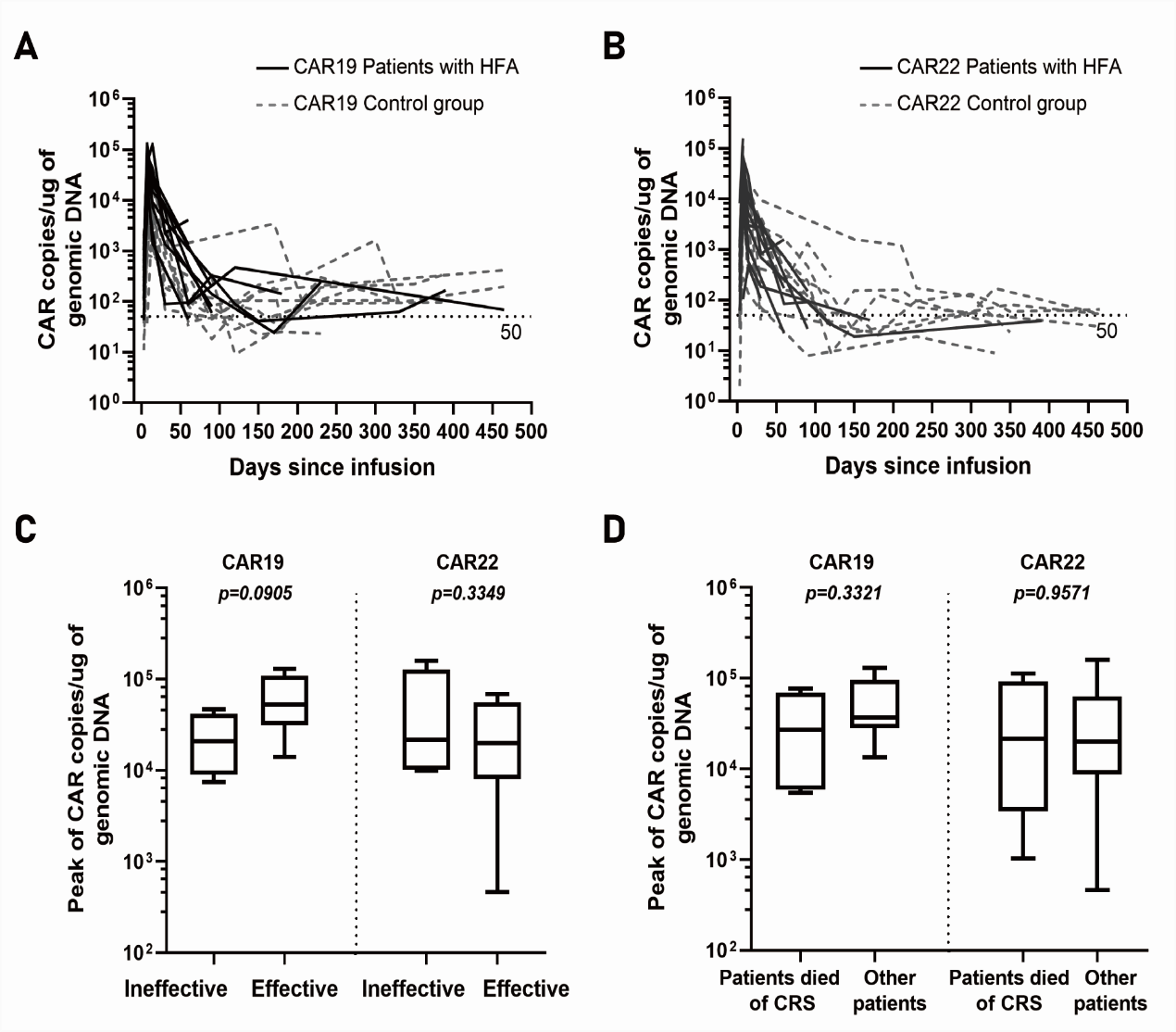


**S Figure 3**. **(A, B)** During the 14 days after CAR T-cell infusion, the medians of the peak values of CAR19 and CAR22 transgene copies in the HFA group were 36767 copies/µg (range, 4770-129375) and 23283.5 copies/µg (range, 464-159171), respectively. In the control group, the medians of the peak values of CAR19 and CAR22 transgene copies were 11393 copies/µg (range, 1255-55774) and 12141 copies/µg (range, 275-159171), respectively. **(C)** The peaks of CAR19 and CAR22 transgenes copies in patients with CR and PR (median 52290.5, range 14118-129375 copies/µg for CAR19 and median 19901.5, range 464-68721 copies/µg for CAR22) were not significantly different with those in nonresponsive patients (median 20755.5, range 7458-46393 copies/µg for CAR19 and median 21507.5, range 9941-159171 copies/µg for CAR22). **(D)** In the HFA group, the patients who died of CRS (n=4) had similar CAR transgene copies to the other patients (n=12) for CAR19 (P=0.3321) and CAR22(P=0.9571).
